# Lessons From Drug Discovery for Cryoprotective Agent Design: An AI‐Oriented Perspective

**DOI:** 10.1002/advs.75947

**Published:** 2026-06-11

**Authors:** Dominika Wilczok, Jesús Valdés‐Hernández, Varinia Bernales, Alán Aspuru‐Guzik, Alex Zhavoronkov

**Affiliations:** ^1^ Duke University Durham North Carolina USA; ^2^ Duke Kunshan University Kunshan Jiangsu China; ^3^ Department of Chemistry University of Toronto Toronto Ontario Canada; ^4^ Acceleration Consortium Toronto Ontario Canada; ^5^ Department of Computer Science University of Toronto Toronto Ontario Canada; ^6^ Vector Institute for Artificial Intelligence Schwartz Reisman Innovation Campus Toronto Ontario Canada; ^7^ Department of Chemical Engineering & Applied Chemistry University of Toronto Toronto Ontario Canada; ^8^ Department of Materials Science & Engineering University of Toronto Toronto Ontario Canada; ^9^ Institute of Medical Science Toronto Ontario Canada; ^10^ NVIDIA Toronto Ontario Canada; ^11^ Canadian Institute for Advanced Research (CIFAR) Toronto Ontario Canada; ^12^ Insilico Medicine US Inc. Cambridge Massachusetts USA; ^13^ Insilico Medicine AI Ltd Abu Dhabi UAE; ^14^ Insilico Medicine Hong Kong Ltd Hong Kong SAR China

**Keywords:** AI‐assisted molecular design, artificial intelligence, cryoprotective agents, engineered cryoprotective materials, multiparameter optimization

## Abstract

Cryopreservation is the storage of biological materials like cells, tissues, or even organs at cryogenic temperatures. This technology is a key enabler for biobanking, reproductive medicine, and cell therapy, and is positioned as a vital part of the future of transplantation. Successful cryopreservation relies on cryoprotective agents (CPAs) that protect biological structures from ice‐induced damage. However, CPAs can have significant drawbacks, including toxicity, particularly at the high concentrations required for vitrification. As efforts advance toward preserving more sensitive cells, whole organs, and, ultimately, entire organisms, there is a pressing need for new CPAs with improved profiles across multiple parameters. The drug discovery discipline has long recognized that an effective compound must meet many criteria beyond potency, absorption, distribution, metabolism, elimination, and toxicity (ADME‐T), and that these criteria must be balanced through multiparameter optimization. Similarly, an ideal cryoprotectant must simultaneously satisfy a broad spectrum of requirements. In this perspective, lessons from drug discovery are applied to the design of cryoprotectants. Treating cryoprotectant development as a multiparameter optimization challenge, akin to drug lead optimization, could enable systematic design of the next generation of safer and more effective CPAs.

## Graphical abstract

Cryoprotectant design is reframed through the lens of drug discovery as a multiparameter optimization problem. This perspective highlights how AI and systematic design strategies could enable safer, more effective cryoprotectants, while identifying key limitations that currently constrain predictive progress in cryobiology.

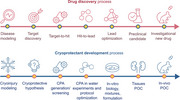

## 1 Introduction

In recent decades, technological revolutions have reshaped the landscape of drug discovery. Advances in analytical methods, the genomic mining of biosynthetic pathways [1], combinatorial and computational chemistry [2, 3], and high‐throughput screening [4, 5] have collectively accelerated the pace at which potential drugs are identified and tested [6]. Today, hundreds of thousands of compounds can be evaluated in automated assays, dramatically expanding the molecular repertoire [7]. Moreover, emerging technologies such as quantum computing and artificial intelligence (AI) promise to further transform this field by enabling unprecedented levels of molecular simulation and optimization, potentially unlocking new therapeutic avenues [8, 9]. Each new technology made chemical space exploration faster, cheaper, and more systematic. A similar convergence could be envisioned for cryoprotectant research: computational chemistry and molecular dynamics could predict how candidate molecules interact with water networks during freezing; automated microfluidic platforms could screen thousands of CPA formulations under controlled cooling conditions, and artificial‐intelligence‐based models could identify patterns linking molecular structure to vitrification efficiency or toxicity. However, such rapid implementation of new technologies has not been applied to develop new CPAs on a large scale. Since the identification of glycerol in 1949 [10] and DMSO in 1959 [11] as effective CPAs, only a handful of alternative substances have been seriously considered [12, 13]. Compared to the vast diversity of molecules explored in pharmacology, this scarcity enables a fertile research direction. CPAs are commonly classified as permeating or non‐permeating based on their ability to cross the cell membrane [14]. Permeating CPAs are small, membrane‐permeable molecules that enter cells and reduce intracellular ice formation by replacing water and lowering the freezing point. Among these, glycerol and dimethyl sulfoxide (DMSO) were the first CPAs to be widely adopted in cryobiology and remain in extensive use today, while ethylene glycol and propylene glycol were introduced later and have since become integral components of many modern cryopreservation protocols. In contrast, non‐permeating CPAs do not penetrate the cell membrane but act extracellularly to induce osmotic dehydration, stabilize cell membranes, and inhibit ice crystal growth [14, 15]. Modern cryopreservation strategies for cells, tissues, and organs use multi‐component ‘cocktails’, such as DP6, VS55, and M22, whose compositions and functions differ in ingredient ratios, additives, or vitrification performance [16, 17]. DP6 is a relatively simple mixture based mainly on DMSO and propylene glycol; VS55 adds formamide to this combination, increasing its glass‐forming ability; and M22 incorporates multiple permeating CPAs (e.g., DMSO, ethylene glycol, formamide) together with polymeric and synthetic ice‐blocking additives, providing superior suppression of ice nucleation and recrystallization. These cocktails are typically combined with non‑permeating sugars and carbohydrates (e.g., trehalose and sucrose) [18, 19], macromolecular colloids (e.g., hydroxyethyl starch and hyaluronic acid) [20, 21], and mechanism‑targeted additives such as antioxidants and other stress modulators (e.g., catalase and ascorbic acid) or ice‑recrystallization inhibitors [22, 23, 24].

The discrepancy between the pace of innovation in drug discovery and in cryoprotectant development highlights a critical gap in translational innovation and raises the question of why modern discovery paradigms have not been systematically applied to the development of next‐generation cryoprotectants. One key distinction lies in the physicochemical context. Drug discovery typically assumes a stable thermodynamic environment, human homeostasis, within which molecular interactions occur. Cryoprotectants, however, operate under radically different conditions. They must function at extremely low temperatures, sometimes under non‐atmospheric pressure, and during dynamic cooling and rewarming processes in which both temperature and pressure vary over time. The performance of a CPA thus depends not only on its molecular properties but also on the physical setup of preservation itself.

While drug development often hinges on processes that can be readily modeled computationally, such as molecular interactions with biological targets, cryopreservation deals with phenomena that are far more complex to simulate. This includes crystal growth, vitrification, and the inhibition of ice formation, all of which involve non‐equilibrium processes that are notoriously difficult to describe [25]. Crystal surfaces may be unstable, giving rise to dendritic growth patterns [26]. Although methods such as phase‐field modelling or cellular automata can capture some of these dynamics [27, 28], they require substantial expertise and computational effort. The behavior of aqueous solutions during crystal growth is inherently difficult to predict, and the addition of CPAs further complicates the picture. CPAs alter macroscopic properties of water and solutions; they shift the chemical potential associated with ice nucleation, increase viscosity, and modulate molecular motion to delay crystal formation [29]. These effects collectively modify cooling rates, sometimes by several orders of magnitude, and influence thermal conductivity, which governs the release of latent heat during freezing.

A systematic investigation of the physicochemical properties of cryoprotectant solutions and their influence on vitrification and crystal growth is, therefore, a fundamental pillar for progress in this field. Just as drug molecules are designed to interact with biological targets in precise and predictable ways, cryoprotectants must be understood and engineered according to their interactions with the thermodynamic and kinetic landscape of water and ice formation. Developing such a mechanistic understanding will be crucial for transforming cryobiology from an empirical practice into a discipline of rational molecular design.

In essence, new cryoprotectant discovery stands today where drug discovery stood a century ago, guided largely by empirical knowledge. The core technological capabilities, such as advanced cryogenic instrumentation, controlled‐rate freezing systems, high‐resolution imaging, and computational modeling, are already available, awaiting the technological and conceptual leap that will make it predictive and design‐driven with the main bottleneck being the lack of publicly available, high‐quality data that would enable a deeper understanding of the cryopreservation‐related phenomena, thereby allowing the design and development of new materials and methodologies.

This work is organized as follows. In the next section, we frame cryoprotectant development explicitly as a multiparameter optimization (MPO) problem, drawing concepts and workflows from modern drug discovery, including target product profiles, lead optimization, and combination strategies. We then formalize the conceptual parallels between drug discovery and cryoprotectant design, establishing a shared vocabulary that maps pharmacological metrics such as potency, safety, and bioavailability onto cryobiological performance criteria. Building on this framework, we next examine in detail the key factors governing cryoprotectant performance, efficacy and mechanism, toxicity and tolerance, delivery and transport, and practical translational constraints, moving progressively from conceptual analogies to physicochemical and biological specificity.

## 2 Multiparameter Optimization in Cryoprotectant Design

Drug discovery teaches us the importance of balancing efficacy with safety and “drug‐like” properties early in development. A promising drug candidate would fail if, for example, it is highly potent but insoluble or toxic [30]. Similarly, a cryoprotectant must do more than simply inhibit ice formation–it must do so in a biocompatible manner that preserves cellular or tissue viability.

Key lessons from drug discovery that inform cryoprotectant design include:

1 **Defining an Ideal Target Profile**: Medicinal chemists often establish a Target Product Profile with desired ranges for potency, solubility, permeability, etc [31]. Similarly, cryobiologists can define a “Target Cryoprotectant Profile” which would comprise a set of ideal properties to maximize preservation outcomes. These include high aqueous solubility at low temperature, good permeation into cells, minimal toxicity, chemical inertness, and stability (as detailed below). This is analogous to Lipinski's Rule of Five in drug design, which sets physicochemical benchmarks for oral drug candidates [32] and could serve as benchmarks for a viable CPA.
2 **Multi‐Objective Screening**: Modern drug discovery often uses high‐throughput screening and computational models to evaluate thousands of compounds against multiple criteria. For example, a study used a deep generative model to propose ≈30 000 kinase inhibitors in 21 days, from which six were synthesized, and potent DDR1 leads were validated, compressing hit identification from months to under two months when counting design, synthesis, and in vitro testing (≈46 days end‐to‐end) [33]. The cryoprotectant discovery field is far from this level of AI application, but it is slowly adopting machine learning approaches. For example, Warren et al. (2024) used machine learning to predict small molecules that inhibit ice recrystallization, screening a library to identify new hits that improved cell survival [34, 35, 36]. Such data‐driven or combinatorial approaches, supported by robotic liquid‐handling platforms that enable simultaneous dispensing of diverse CPA concentrations across multi‐well formats, can rapidly identify candidates that would be hard to find by trial‐and‐error alone, moving beyond the historical reliance on a few known CPAs.
3 **Iterative Lead Optimization**: In medicinal chemistry, initial “hits” are systematically optimized for better ADME‐T properties while retaining efficacy. Analogously, one might start with a known cryoprotective molecule (like a glycol or amide) and modify its structure to reduce toxicity or improve permeability. This might entail adding or removing functional groups to tune hydrogen bonding and lipophilicity–much as one would adjust a drug molecule to improve cell membrane crossing or reduce off‐target binding. The development of “cryoprotectant analogues” (for instance, formamide analogues combined with DMSO in vitrification solutions) reflects this kind of rational modification.
4 **Combination Therapies**: Just as drug cocktails can provide synergistic effects (e.g., multi‐drug regimens in HIV or cancer therapy to balance efficacy and toxicity [37, 38, 39]), cryoprotection often employs mixtures of CPAs to leverage their complementary strengths. In fact, combining cryoprotectants is a proven strategy to reduce toxicity while maintaining or enhancing ice inhibition [40]. Multi‐component solutions, or CPA cocktails, are designed in a manner analogous to combination drug therapy, achieving synergistic effects that exceed the sum of their individual contributions.

By applying the same rigor and creativity used in drug discovery, researchers can systematically design cryoprotectants with optimized efficacy, safety, and practicality. The following sections detail the critical parameters governing cryoprotectant performance and highlight parallels to modern drug discovery.

## 3 Conceptual Equivalences Between Drug Discovery and Cryoprotectant Design

**FIGURE 1:**
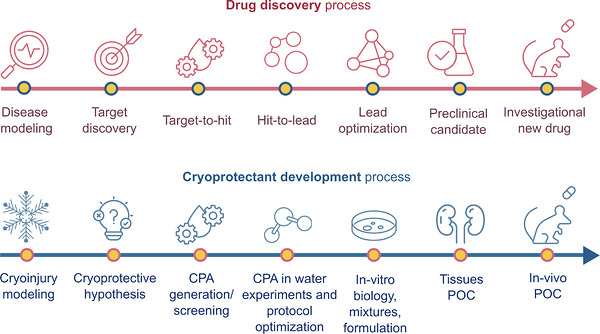
Parallels between the drug discovery pipeline and our proposed cryoprotectant design pipeline.

A useful way to structure cryoprotectant discovery is to draw direct analogies with the well‐established paradigms of drug discovery. Both fields face the challenge of balancing multiple, often conflicting parameters: potency with safety, delivery with clearance, manufacturability with cost. In drug discovery, these challenges have been formalized into “golden rules” such as Lipinski's Rule of Five [41], therapeutic index calculations [42], and the ADME‐T framework [43]. Cryoprotectant research is still maturing toward such codified benchmarks, but clear parallels can already be drawn. Figure 1 outlines the steps in early drug discovery, and their proposed equivalent in cryoprotectant design.

Table 1 summarizes these conceptual equivalents. Multiparameter optimization tools can be imported from drug discovery and evaluation frameworks in pharmacology and applied directly to the design of new CPAs. For example, what pharmacologists quantify as “bioavailability” has its counterpart in the permeability and tissue distribution of a CPA; the “therapeutic index” corresponds to the maximum tolerated concentration and exposure time; and combination therapy mirrors multicomponent CPA cocktails.

**TABLE 1:** Parallels between drug discovery and cryoprotectant design.

Drug discovery golden standard	Cryoprotectant discovery equivalent
Potency (IC50, EC50)–the drug must be effective at a reasonable concentration	Cryoprotective efficacy–ability to prevent ice formation, vitrify, or inhibit recrystallization at achievable concentrations
Safety/Therapeutic Index–margin between effective and toxic dose	Tissue‐specific tolerance window–maximum tolerated concentration & exposure time before cytotoxicity
ADME (Absorption, Distribution, Metabolism, Excretion)–determines bioavailability	Spatial and temporal CPA exposure across cellular and extracellular compartments–determined by permeability, diffusivity, viscosity, and delivery strategy
Lipinski's Rule of Five–physicochemical heuristics for oral drug‐likeness	Proposed cryo's Rule of Five (*this work*)–size, polarity, H‐bonding, solubility thresholds for CPA permeation, and water miscibility
Pharmacokinetics (PK)–time‐course of drug levels in body compartments	Loading/Unloading Kinetics–CPA equilibration and removal time, osmotic fluxes during protocols
Pharmacodynamics (PD)–molecular target engagement and effect	Protection Mechanism–colligative effect, vitrification, ice recrystallization inhibition (IRI) activity, membrane/protein stabilization
QSAR/Structure‐Activity Relationships–relate molecular structure to potency/toxicity	QSAR/Structure–Property Models for Cryo–relate chemical features to glass transition temp, toxicity, permeability
High‐Throughput Screening–rapid testing of compound libraries	High‐Throughput Screening/Microfluidic Assays–rapid screening of CPA mixtures for viability, vitrification, IRI activity
Therapeutic Index Optimization–balance between efficacy and safety	Multiparameter Optimization (MPO)–balance between vitrification efficiency, toxicity, solubility, viscosity
Combination Therapy–drugs combined for synergy and reduced side effects	CPA Cocktails–combination of penetrating, non‐penetrating, and polymeric CPAs to maximize protection, minimize toxicity
Drug Formulation & Solubility Rules–ensure the drug can be delivered at the needed dose	Solution Stability & Solubility–CPA miscibility, low‐temperature solubility, viscosity manageable for perfusion
Biomarker‐based Safety Assessment–genomics, toxicogenomics	Cell/Tissue Viability Metrics–LD50, post‐thaw metabolic activity, apoptosis markers
Regulatory Benchmarks (FDA, EMA)–approval standards	Benchmark Protocols (e.g., ISBER, GMP cryomedia)–standardized testing for clinical adoption
Patient Variability–pharmacogenomics, comorbidities	Tissue Variability–differences in CPA tolerance between cell types and organs

## 4 Key Parameters for an Optimal Cryoprotectant

Designing an optimal CPA requires balancing multiple interdependent factors, such as maximizing ice‐blocking efficacy, minimizing toxicity, ensuring efficient tissue‐scale delivery, and achieving practical manufacturability and regulatory feasibility. The following sections outline the key parameters that define successful cryoprotectant performance, including biophysical mechanisms and safety constraints, transport kinetics, and considerations for real‐world translation.

### 4.1 Efficacy & Mechanisms

The functional analogue of drug “potency” is the ability of a CPA to prevent ice crystal formation and preserve the sample's function. Penetrating CPAs such as DMSO, glycerol, ethylene glycol, and propylene glycol act primarily through colligative effects, lowering the chemical potential of water and thereby depressing the equilibrium freezing point. This reduces the thermodynamic driving force for ice nucleation and growth, stabilizing the liquid in a metastable supercooled state [44]. They also disrupt hydrogen bonding within the aqueous phase, increasing the kinetic barrier to ice crystal formation; at sufficiently high concentrations and cooling rates, these combined effects promote vitrification rather than crystallization [45, 46]. Efficacy can therefore be understood in terms of the glass transition temperature and the critical cooling and warming rates required for an ice‐free trajectory at specific concentrations [47]. Ice recrystallization inhibition (IRI) is a complementary efficacy axis most relevant during rewarming and transient holds. Ice‐active additives, such as poly(vinyl alcohol) (X‐1000) and polyglycerol (Z‐1000), are incorporated into organ vitrification cocktails, such as M22, to suppress ice nucleation and growth and reduce the risk of devitrification during rewarming [48, 49]. Non‐penetrating sugars and polymers (e.g., trehalose, sucrose, hydroxyethyl starch) increase solution viscosity and elevate the glass transition temperature of the unfrozen extracellular fraction. These effects reduce molecular mobility, thereby suppressing diffusion‐driven damage and limiting degradative chemical reactions during cooling and storage [50]. In parallel, disaccharides such as trehalose and sucrose exert protective effects through water‐replacement and preferential hydration mechanisms: they hydrogen‐bond with lipid headgroups and protein surfaces, stabilizing membrane phase behavior and preserving native protein conformations under dehydration and low‐temperature stress [51, 52].

### 4.2 Safety & Tolerance

Efficacy is constrained by safety at two levels: cellular toxicity during exposure and systemic risk posed by residual products destined for infusion or implantation. However, they are multifaceted and not fully understood. For instance, reports on the extent and nature of DMSO‐induced damage are conflicting, depending on concentration, exposure time, tissue type, and whether the sample was cryopreserved [11, 53]. This ambiguity might be contributing to the slow development of new cryoprotectants; as described in Figure 1, the cryoinjury (here, cryoprotectant toxicity pathways) is not well mapped, therefore, the subsequent development stages cannot follow. In contrast, non‐penetrating cryoprotectants exhibit low intrinsic toxicity, but because they remain extracellular, abrupt changes in their concentration can impose damaging osmotic stress during loading or removal [54].

### 4.3 Delivery & Distribution

Successful cryopreservation and rewarming depend not merely on whether a CPA permeates but on the rate of permeation: too slow permeation invites shrinkage‐induced injury during loading; too fast risks swelling on removal. Introducing non‐penetrating protectants may require temporary membrane permeabilization or transporter‐mediated strategies; microfluidic mixing can shorten exposure by accelerating equilibration at controlled temperatures.

CPAs should be highly water‐miscible and remain in a single phase upon cooling; otherwise, precipitation or phase separation undermines delivery. Low‐temperature miscibility and resistance to crystallization favor glass formation [55]. Elevated viscosity, although beneficial for preventing ice formation, hinders perfusion and mixing, compromises uniform distribution, and can complicate rewarming by slowing flow in devitrifying matrices [56]. Practical solutions include selecting less viscous penetrating agents and using pressure‐assisted perfusion while recognizing that mechanical injury from excessive flow or pressure may become more limiting than intrinsic CPA cytotoxicity.

Each tissue exhibits a distinct cryoprotectant tolerance window (concentration, exposure time, temperature) [57] set jointly by vascular perfusion dynamics and cell‐level membrane transport. At the organ scale, CPA loading and washout are governed by mass transfer limits, vascular architecture, and barrier properties along the path from circulation to cells (distribution through the vascular tree, transvascular exchange, diffusion through interstitium/extracellular matrix, and finally membrane transport); because the relevant physiologic, geometric, and transport parameters differ among tissues, their CPA requirements necessarily diverge [58]. For organ‐scale perfusion, diffusion limits and regional flow disparities further tighten tolerability. At the cellular scale, membrane lipid composition modulates CPA–bilayer interactions and permeability [59], just as the number and arrangement of aquaporin and aquaglyceroporin channels [60] determine water and small‐solute CPA fluxes (e.g., glycerol), thereby shaping osmotic‐volume kinetics and protocol tolerances. Operationally, the maximum tolerated dose (MTD) and toxic exposure time (TET) should be specified per tissue, embedded into loading and unloading schedules and multiparameter optimization scoring, transforming “toxicity” from a global scalar into a tissue‐contextual constraint that co‐optimizes with permeability and efficacy.

### 4.4 Practical Translation

Manufacturability, cost, shelf stability, and regulatory acceptability determine whether an optimized CPA profile can reach practice. Clinical and industrial use requires high‐purity liters of solution; simple, widely available chemicals such as DMSO and glycerol set a practical baseline, while proteinaceous or bespoke polymeric agents may impose cost and supply constraints unless bioengineered or synthesized at scale with reproducible molecular weight distributions. Chemically defined, xeno‐free formulations with long refrigerated shelf‐lives are preferred for consistency, and Good Manufacturing Practices (GMP), the regulatory system guidelines encompassing standardized manufacturing processes, quality control, documentation, and traceability required for clinical‐grade biomedical products, should be followed. Regulatory pathways treat CPAs as ancillary reagents or components of final products–although this regulation may change as organ or organismal cryopreservation enters the market–so established safety profiles (e.g., GRAS‐like status, prior clinical use) can facilitate adoption. In contrast, novel scaffolds demand extensive toxicological evaluation and regulatory clearance.

The development engine should mirror the Design–Make–Test–Analyze loop of drug discovery [61]. Structure–property modeling (‘cryo‐QSAR’) uses statistical links between molecular structure and properties to prioritize scaffolds for glass transition temperature, permeability, toxicity, and viscosity; high‐throughput microfluidic assays and differential scanning calorimetry can screen mixtures for vitrification windows and IRI potency, while machine learning integrates outcomes to propose next‐round compositions along Pareto fronts, which are the set of optimal trade‐offs that balance efficacy against exposure and handling constraints. Benchmarking remains a field‐level gap; standardized assays for viability, function, vitrification efficiency, warming robustness, and residual‐CPA effects across tissues would enable transparent comparison analogous to pharmacology's common potency and safety readouts.

## 5 Cryopreservation's Materials Challenges and Outlook

Ultimately, the search for an ideal cryoprotectant is not only a biochemical problem but also a profound materials challenge. The dominance of water in biological systems makes cryopreservation fundamentally a problem of controlling phase transitions and solid‐state behavior of this liquid under extreme thermodynamic stress. The emerging view is that advancing the field will require moving beyond purely empirical formulations and toward a mechanistic, materials‐science‐driven understanding of how solutes modulate ice structure, glass stability, and thermal transport across scales. In cryopreservation, vitrification, the transformation of a liquid into a glassy, non‐crystalline solid, effectively inhibits the formation of ice crystals, particularly dendritic ones that can damage cellular structures [62, 63, 64]. However, vitrification represents a metastable state: once achieved, it remains highly susceptible to devitrification and the subsequent growth of dendritic crystals.

If dendritic growth is the main source of damage, it might be possible to design CPAs that would inhibit these specific crystal morphologies. Such agents could enable the controlled formation of non‐damaging crystalline ice structures, avoiding vitrification altogether. With this in mind, CPAs could be conceptually classified into three broad categories according to the solid‐state outcomes they promote:

1. **Anti‐dendritic CPAs**, which suppress the formation of branched crystal structures;
2. **Semi‐amorphous CPAs**, which lead to partially ordered, less damaging ice forms, and
3. **Vitrifying CPAs**, which lower the cooling rate necessary to achieve full vitrification.

**FIGURE 2:**
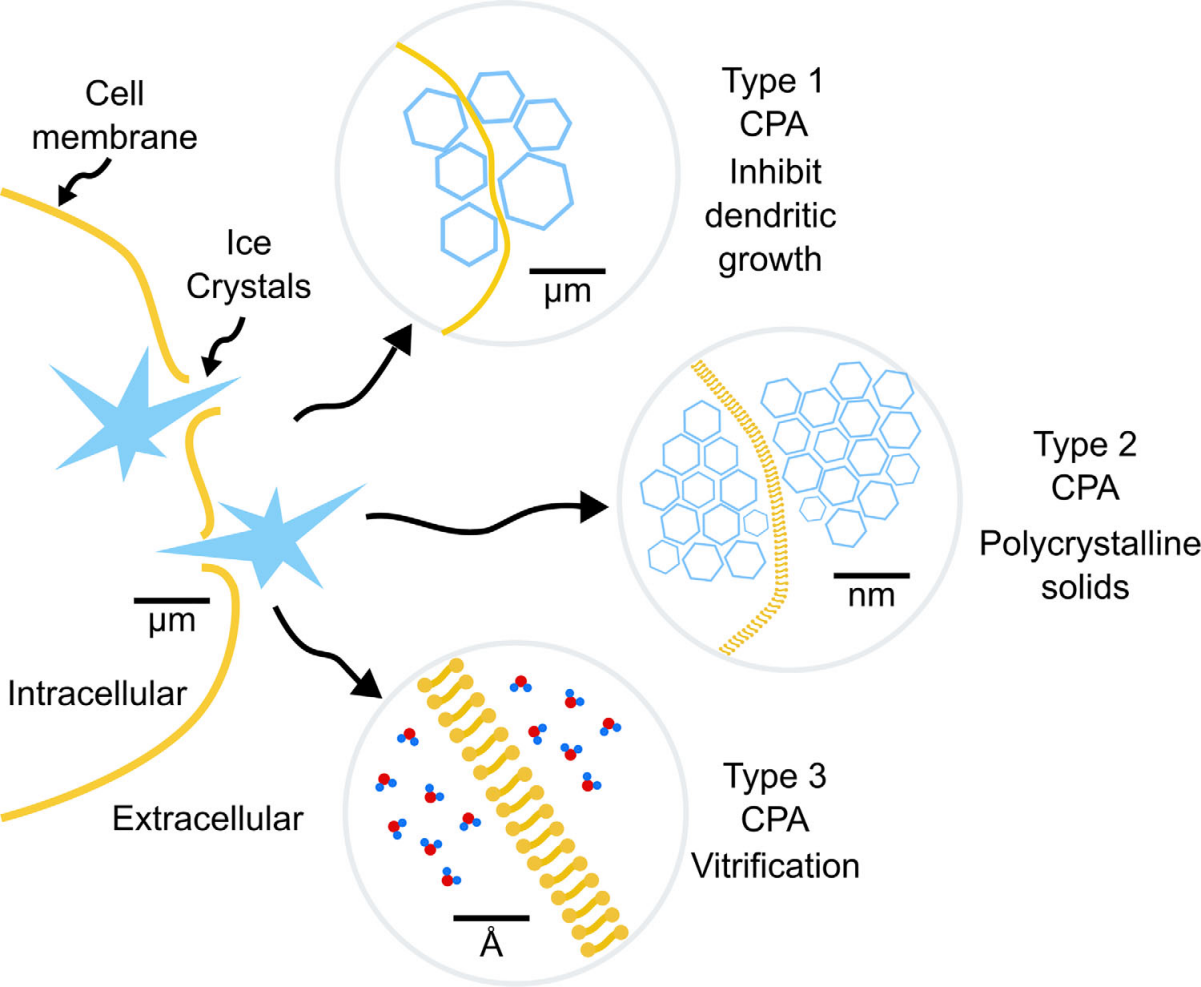
Classification of CPAs according to the type of solid morphologies they can generate. Type 1 CPAs inhibit dendritic growth, which can rupture cellular membranes; as a result, high concentrations of different chemical species accumulate outside the crystals. Type 2 CPAs generate a polycrystalline solid composed of nanometer‐sized crystallites; the concentrations of different chemical species are lower due to the presence of grain boundaries, where these species may be located. Type 3 CPAs promote the formation of amorphous solids.

This classification is not strict; certain compounds may simultaneously inhibit dendritic growth and promote semi‐amorphous ice formation, as exemplified in Figure 2. Micrometric structures formed during ice crystal growth generate mechanical stresses on cell walls, which can rupture the plasma membrane and lead to cell death or the malfunction of intracellular organelles. In addition, changes in solute concentration outside the ice create chemical microenvironments unfavorable for cellular survival [65, 66]. Although much current research focuses on achieving vitrification, it is also important to explore strategies that allow the formation of “benign” solid structures with crystallites small enough to avoid damaging biological components, and with a high density of grain boundaries [67]. These boundaries, lacking a well‐defined crystalline order, can limit the localized concentration increases associated with crystal growth and thereby reduce osmotic damage. Furthermore, the presence of numerous grain boundaries inhibits the formation of dendritic structures, which are responsible for most of the mechanical stresses exerted on cells during freezing.

Another major challenge in CPA discovery lies in the scalability of the freezing and warming protocol. Most cryopreservation protocols have been optimized for small volumes, where thermal conductivity and concentration gradients can often be neglected [68]. However, in larger systems like organs, ignoring these effects becomes problematic. Temperature and solute gradients create local variations in thermodynamic state, which, in turn, alter CPA performance across the sample. This issue is fundamentally rooted in the way current technologies achieve cooling and thawing, primarily through thermal conduction. When a solid phase forms, heat is extracted from the surface outward, leading to temperature gradients. These temperature gradients, together with the concentration gradients near the surface of ice crystals, lead to potentially hazardous dendritic growth [26]. Although electromagnetic radiation sources such as infrared emitters can assist with rewarming, they may not penetrate uniformly, depending on the system's absorption properties at the selected wavelength. This can induce non‐uniform heating, introducing additional spatial variability [69, 70]. A promising approach to achieving uniform heating and suppressing crystal formation is the use of magnetic nanoparticles [71, 72]. When oscillating magnetic fields are applied, these nanoparticles generate heat through magnetic hysteresis losses. This effectively turns the system into a volumetric heat source that, ideally, exhibits minimal temperature gradients, thereby inhibiting the formation of dendritic structures arising from surface instabilities. These technical considerations highlight the need for rigorously standardized and physically consistent experimental platforms for CPA evaluation.

A robust framework for evaluating cryoprotectants should minimize dependence on the experimental setup. This requires standardized procedures, precise control of thermodynamic variables, and a clear understanding of the physical principles governing CPA behavior. Once a CPA is equilibrated, protocols typically proceed through controlled cooling, maintaining the specimen at the target temperature, controlled rewarming, and CPA unloading. The design of these stages, including the rate and timing of temperature ramps, depends on both the biological sample and the CPA formulation. Quantitative measurements of the physicochemical properties of CPAs remain scarce. Data at cryogenic temperatures are often unavailable, and studies often report only qualitative outcomes: whether a CPA cocktail was “successful” at properly vitrifying the specimen and withstanding rewarming without recrystallization. To make cryoprotectant discovery genuinely data‐driven and predictive, aligned with modern drug discovery, we need systematic, standardized, and openly shared measurements across the relevant temperature ranges. As in drug discovery, where machine learning and AI rely on vast, high‐quality datasets, progress in cryobiology will depend critically on the systematic generation and sharing of reliable physicochemical data.

## 6 Considerations and Limitations of AI in Cryoprotectant Design

Application of AI to cryobiology presents distinct methodological challenges that must be explicitly addressed to ensure robust and generalizable outcomes. While recent advances in foundation models and generative AI have shown promise in chemistry and molecular design, these approaches come with their own limitations, including issues related to data quality, domain‐specific generalization (especially to non‐ambient or cryogenic conditions), interpretability, and physical validity of generated structures, as thoroughly discussed in several recent publications [73, 74]. A primary limitation lies in data availability and quality. Unlike drug discovery, where large, standardized datasets exist for molecular properties and biological activity, CPA research remains constrained by sparse, heterogeneous, and often non‐standardized datasets. As highlighted throughout this perspective, many studies report qualitative outcomes (e.g., “successful vitrification”) rather than quantitative physicochemical or biological metrics across controlled conditions. This scarcity introduces a high risk of overfitting, particularly for flexible models such as deep neural networks, which may capture dataset‐specific artifacts rather than generalizable structure–property relationships. Moreover, machine learning models trained on a narrow chemical space, dominated by a small number of classical cryoprotectants such as DMSO, glycerol, and glycols, may fail when extrapolated to novel chemical scaffolds. Predictions outside the training distribution may appear plausible but lack physical validity, especially given the non‐linear and non‐equilibrium phenomena governing cryopreservation, including vitrification kinetics, ice nucleation, and transport‐limited toxicity. Cross‐validation within limited datasets is insufficient; external validation across independent experiments is necessary to establish generalizability.

The selection of molecular descriptors and representations further constrains model performance. Conventional cheminformatics descriptors optimized for drug‐like molecules may not adequately capture properties critical to cryoprotection, such as hydrogen‐bond network modulation, glass transition behavior, or viscosity at sub‐zero temperatures [75]. Incorporating physically informed descriptors, or hybrid approaches combining molecular dynamics simulations with learned representations, may be necessary to bridge this gap.

Recent advances in foundation models and generative AI offer a promising route to overcome some of these limitations by enabling exploration of chemical space beyond known cryoprotectants [76, 77]. However, existing foundation models are typically trained on ambient‐condition chemistry and pharmacological objectives; their learned representations may not encode the thermodynamic and kinetic constraints relevant to cryogenic systems, reinforcing the need to create a standardized CPA dataset with multiple parameters across conditions. One potential avenue to address data scarcity is the use of synthetic data generated from physics‐based simulations. Molecular dynamics, Monte Carlo methods, and phase‐field models can, in principle, provide estimates of properties such as diffusivity, hydrogen‐bond dynamics, or ice nucleation behavior under controlled conditions. These simulated datasets can be used to pretrain or augment machine learning models, effectively expanding the available training space. A promising strategy is multi‐fidelity modeling, in which low‐cost, lower‐accuracy simulations are combined with high‐quality experimental data to guide model training. Active learning frameworks can further improve efficiency by selectively querying experiments in regions of chemical space where model uncertainty is highest.

While foundation models and generative AI may help expand the search beyond known cryoprotectants, most such models are trained on ambient‐condition chemistry and objectives unrelated to cryogenic thermodynamics. Their latent spaces, therefore, may not reflect the constraints that dominate cryopreservation performance, reinforcing the need for standardized CPA datasets spanning temperature, concentration, exposure time, and mixture composition [78, 79]. A pragmatic path forward is multi‐fidelity learning, where synthetic data from physics‐based simulations (molecular dynamics for transport/H‐bonding proxies; continuum models for diffusion‐limited loading; thermal models for cooling/warming constraints) augment limited experiments, and where experimental campaigns are driven by active learning to prioritize measurements in high‐uncertainty or high‐value regions—an approach already shown to be effective for data‐efficient CPA cocktail discovery in multi‐objective Bayesian optimization loops [80]. In parallel, “digital twin” style hybrid modeling efforts that evaluate large numbers of candidate thermal histories in silico illustrate how mechanistic simulation can expand protocol coverage even when experiments are expensive [56, 81, 82].

## 7 Conclusions

Cryoprotectant discovery benefits directly from drug discovery's multiparameter optimization mindset. By treating efficacy mechanisms, safety/tolerance, delivery/distribution, and practical translation as interdependent axes, and by embedding tissue‐specific tolerance windows and transport physics into design, new CPAs and cocktails can be engineered to vitrify at feasible concentrations, minimize toxicity, and perform under clinically realistic loading and warming schedules. The dramatic acceleration of drug discovery through high‐throughput screening, computational chemistry, and AI highlights what cryobiology has largely lacked: systematic exploration of chemical space and materials science coupled with rigorous, quantitative datasets. With cryo‐QSAR, high‐throughput experimentation, and rigorous benchmarking, this science‐first framework can expand the CPA toolbox, enabling robust, scalable preservation from cells to organs.

Viewing CPAs through a materials‐science lens further broadens the design space. Because cryoinjury arises from phase transitions and solid‐state dynamics, promising strategies include not only improved vitrifying agents but also CPAs that inhibit dendritic ice growth or promote benign semi‐amorphous structures. Addressing scalability challenges in tissues and organs will require controlling thermal gradients during rewarming; approaches such as magnetic nanoparticle‐mediated volumetric heating exemplify how physical innovations can complement chemical design. Collectively, these developments argue for a science‐first framework in which CPA behavior is benchmarked across the relevant thermodynamic landscape and captured in openly shared datasets that can train predictive machine‐learning models.

Translating lessons from drug discovery reframes CPA design as a multi‐objective optimization problem with explicit constraints on safety, transport, phase behavior, and practical deployability. The most promising paths couple rational mixture design and transport‐aware protocols with scalable, biocompatible chemistries and standardized benchmarks.

## Author Contributions

Dominika Wilczok: writing ‐ orginal draft, visualization, writing – review and editing. **Jesus Valdes‐Hernandez**: writing – original draft, visualization, writing – review and editing. **Varinia Bernales**: writing – review and editing. **Alan Aspuru‐guzik**: writing – review and editing. Alex Zhavoronkov: writing – original draft, conceptualization, writing – review and editing.

## Conflicts of Interest

A.Z. is affiliated with Insilico Medicine, a commercial company that develops and utilizes AI platforms for therapeutic target discovery, drug repurposing, and indication expansion. A.A.‐G, D.W., J.V.H, and V.B. declare no competing interests.

## Data Availability

Data sharing not applicable to this article as no datasets were generated or analyzed during the current study.
